# Anchoring genome sequence to chromosomes of the central bearded dragon (*Pogona vitticeps*) enables reconstruction of ancestral squamate macrochromosomes and identifies sequence content of the Z chromosome

**DOI:** 10.1186/s12864-016-2774-3

**Published:** 2016-06-10

**Authors:** Janine E. Deakin, Melanie J. Edwards, Hardip Patel, Denis O’Meally, Jinmin Lian, Rachael Stenhouse, Sam Ryan, Alexandra M. Livernois, Bhumika Azad, Clare E. Holleley, Qiye Li, Arthur Georges

**Affiliations:** Institute for Applied Ecology, University of Canberra, Canberra, ACT 2601 Australia; John Curtin School of Medical Research, Australian National University, Canberra, ACT 2601 Australia; China National GeneBank, BGI-Shenzhen, Shenzhen, 518083 China; Centre for GeoGenetics, Natural History Museum of Denmark, University of Copenhagen, Øster Voldgade 5-7, Copenhagen, 1350 Denmark

**Keywords:** Cytogenetic map, Genome evolution, Ancestral karyotype, Reptile, Macrochromosome, Microchromosome, Sex chromosome, Sex determination

## Abstract

**Background:**

Squamates (lizards and snakes) are a speciose lineage of reptiles displaying considerable karyotypic diversity, particularly among lizards. Understanding the evolution of this diversity requires comparison of genome organisation between species. Although the genomes of several squamate species have now been sequenced, only the green anole lizard has any sequence anchored to chromosomes. There is only limited gene mapping data available for five other squamates. This makes it difficult to reconstruct the events that have led to extant squamate karyotypic diversity. The purpose of this study was to anchor the recently sequenced central bearded dragon (*Pogona vitticeps*) genome to chromosomes to trace the evolution of squamate chromosomes. Assigning sequence to sex chromosomes was of particular interest for identifying candidate sex determining genes.

**Results:**

By using two different approaches to map conserved blocks of genes, we were able to anchor approximately 42 % of the dragon genome sequence to chromosomes. We constructed detailed comparative maps between dragon, anole and chicken genomes, and where possible, made broader comparisons across Squamata using cytogenetic mapping information for five other species. We show that squamate macrochromosomes are relatively well conserved between species, supporting findings from previous molecular cytogenetic studies. Macrochromosome diversity between members of the Toxicofera clade has been generated by intrachromosomal, and a small number of interchromosomal, rearrangements. We reconstructed the ancestral squamate macrochromosomes by drawing upon comparative cytogenetic mapping data from seven squamate species and propose the events leading to the arrangements observed in representative species. In addition, we assigned over 8 Mbp of sequence containing 219 genes to the Z chromosome, providing a list of genes to begin testing as candidate sex determining genes.

**Conclusions:**

Anchoring of the dragon genome has provided substantial insight into the evolution of squamate genomes, enabling us to reconstruct ancestral macrochromosome arrangements at key positions in the squamate phylogeny, demonstrating that fusions between macrochromosomes or fusions of macrochromosomes and microchromosomes, have played an important role during the evolution of squamate genomes. Assigning sequence to the sex chromosomes has identified *NR5A1* as a promising candidate sex determining gene in the dragon.

**Electronic supplementary material:**

The online version of this article (doi:10.1186/s12864-016-2774-3) contains supplementary material, which is available to authorized users.

## Background

Genome assemblies need to be anchored to chromosomes if they are to be useful for addressing important questions in genome evolution [1]. The time consuming and costly exercise of developing a chromosomal based assembly is often overlooked yet the benefits gained from an anchored genome are immense. Knowledge of how genomes have evolved provides an understanding of the role genome organisation plays in the evolution of species, including the evolution of sex determining genes. Tracing genome evolution is achieved by comparing genome organisation between species to reconstruct the most likely chromosome arrangement in a common ancestor.

Until recently, ancestral karyotype reconstructions depended largely on cross-species chromosome painting, which enabled the prediction of ancestral karyotypes for eutherian mammals [2] and avian macrochromosomes [3]. Ancestral karyotype reconstructions over greater evolutionary distances are possible when chromosome painting data are combined with gene mapping and whole genome sequence data. For instance, a comparison of gene mapping data for the tammar wallaby (*Macropus eugenii*) genome compared with anchored genome assemblies for the grey short-tailed opossum (*Monodelphis domestica*), chicken (*Gallus gallus*) and human permitted the first prediction of the ancestral therian (marsupial and eutherian) mammal karyotype [4]. Similarly, a comparison of gene mapping data for species of turtle, crocodile, frog, salamander and snake compared with genome assemblies for chicken and human enabled the ancestral karyotype of amniotes to be predicted [5]. Despite the prediction of the amniote protokaryotype, there are still key amniote lineages for which there are gaps in our understanding of chromosome evolution.

Reptiles, excluding birds, number some 10,000 species and present an excellent group in which to study chromosome evolution, since they display a high level of diversity in chromosome number and morphology, in the absence or presence of microchromosomes, and diversity in sex determination systems (genetic or temperature dependent) and sex chromosomes (reviewed in [6]). Squamates (snakes and lizards) show a high level of karyotypic diversity, with diploid chromosome numbers ranging from 24 to 50, yet are an understudied lineage for detailed investigations into karyotypic changes. In the past, comparisons between squamate species have been limited to global levels of homology between species within the Scinidae (skinks) [7] and Gekkonidae (gekkos) [8]. More broad based studies determined homology among nine families (10 species) of squamates using four chicken chromosome paints (chromosomes 3, 5, 7 and Z), revealing strong conservation of these chromosomes among the ten species [9, 10]. Cytogenetic maps, providing the location of specific genes or DNA clones on chromosomes, are available for one species of snake (Japanese four-striped snake - *Elaphe quadrivirgata*) and six species of lizards (central bearded dragon - *Pogona vitticeps* [11], water monitor lizard - *Varanus salvator macromaculatus* [12], savannah monitor lizard - *V. exanthematicus* [12], butterfly lizard - *Leiolepis reevesii* [13], sand lizard - *Lacerta agilis* [14] and Hokou gecko - *Gekko hokouensis* [15]), - albeit the *V. exanthematicus* map is limited to just 17 genes. Five squamate genomes (green anole lizard *Anolis carolinensis*, Burmese python - *Python molurus bivittatus*, king cobra - *Ophiophagus hannah*, Asian glass lizard - *Ophisaurus gracilis,* Schlegel’s Japanese Gecko – *Gekko japonicus* [16–20]) have been previously sequenced but only the anole has any sequence anchored to chromosomes (i.e. there is no information on the chromosomal location of sequence for the two snakes, gecko or the Asian glass lizard), making it impossible to determine genome rearrangements using these sequenced species. The recently anchored painted turtle genome (*Chrysemys picta*) has highlighted the finer scale resolution afforded by combining cytogenetic mapping with genome sequence assemblies. Unlike squamates, turtle karyotypes are typically highly conserved [21, 22]. The anchored turtle genome assembly uncovered many chromosomal rearrangements, challenging the previously held view of a high level of macrochromosome conservation between birds and turtles [23].

Squamates also display diversity in sex determination systems, varying between genetic sex determination (GSD) and temperature dependent sex determination (TSD) or even an interaction between these two systems [24, 25], with a transition from GSD to TSD recently reported in captive populations of dragon (*P. vitticeps*) [26]. In squamates with GSD, independent evolution of sex chromosomes has occurred in different lineages. This is demonstrated by using chicken as a reference species: gecko Z chromosome shares homology with the chicken Z [27]; the sand lizard Z shares homology with chicken chromosomes 6 and 9 [14]; snake Z genes correspond to those on chicken chromosomes 2 and 27 [28–30]; the anole X chromosome shares homology with chicken 15 [18, 31]. Two genes on chicken chromosome 23 have been mapped to the dragon Z chromosome*,* although another chicken 23 gene maps to an autosomal microchromosome in this species, so it cannot be assumed that the entire Z chromosome shares homology with chicken 23 [32]*.* With the exception of the anole*,* where 250 genes have been assigned to the X [31], and snakes, where genes were assigned to the Z based on genome sequencing or quantitative PCR [30], fewer than 10 genes have been mapped to squamate sex chromosomes [13–15, 29, 32, 33]. A greater understanding of sex chromosome evolution in squamates requires more genes to be assigned to their sex chromosomes.

The genome of the central bearded dragon, a squamate from the family Agamidae, has recently been sequenced and assembled into 545,310 sequence scaffolds [34]. The dragon has a karyotype consisting of six pairs of macrochromosomes and ten pairs of microchromosomes (including ZZ or ZW sex chromosomes) [35, 36]. Most importantly, considerable resources for this species are available to enable this genome assembly to be anchored to chromosomes, including a bacterial artificial chromosome (BAC) library as a source of probes for molecular cytogenetic mapping and a preliminary cytogenetic map consisting of 87 BAC clones [11]. An anchored genome sequence for a second squamate, along with adequate cytogenetic mapping data for five other species, provides the opportunity to reconstruct the ancestral chromosome arrangements at key points in squamate evolution.

The purpose of this study was to anchor dragon sequence to chromosomes, including the Z chromosome, in order to more accurately determine the level of rearrangement between squamates, and more broadly, to trace the evolution of squamate genomes. To accomplish this task efficiently, we built on the existing dragon cytogenetic map [11] by employing a strategy of cytogenetically mapping conserved anole-chicken and chicken-human gene blocks, assembling super-scaffolds based on conserved synteny and constructing comparative maps for dragon, anole and chicken genomes. We compared these maps to the more limited gene mapping data available for five other species [12, 14, 15, 29] to determine the make-up of ancestral squamate macrochromosomes. We have gained a greater understanding of the composition of dragon microchromosomes, including the Z, and have identified a promising candidate sex determining gene.

## Results and discussion

We used two different approaches to map large, conserved blocks of genes to the six macrochromosomes and 10 microchromosomes of the dragon*.* With the first approach, we isolated BAC clones containing genes located at the ends of either anole-chicken or human-chicken homologous synteny blocks. By assembling super-scaffolds based on conserved synteny analysis we then further extended the amount of sequence assigned to dragon chromosomes. Comparative maps were then constructed by comparing the location of sequence on dragon chromosomes to that in the chicken and anole genome assemblies, enabling us to begin tracing the evolution of squamate chromosomes and determine the sequence content of the dragon Z chromosome.

### Assignment of genome sequence to dragon autosomes

**Fig. 1:**
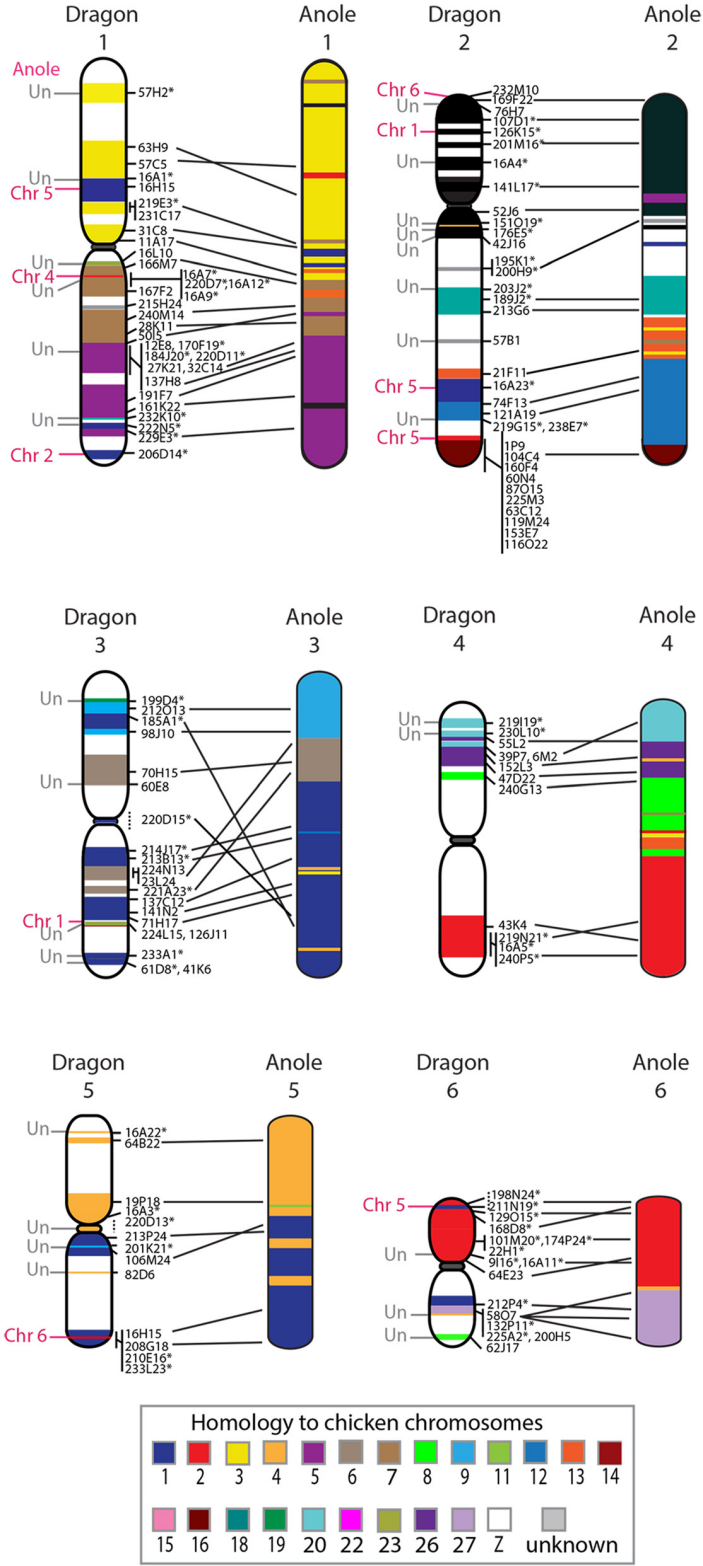
Cytogenetic map of dragon macrochromosomes and dragon-anole comparative map. The position of BAC clones is indicated on the *right* of each dragon chromosome. BACs mapped on the first generation cytogenetic map [11] are indicated by an *asterisk*. Chromosomes are colour-coded for their homology to chicken chromosomes. Lines between dragon and anole chromosomes indicate the relative position on the anole chromosome of the dragon scaffold or super-scaffold anchored by each BAC clone. Interchromosomal rearrangements are indicated to the *left-side* of the dragon chromosome with the anole chromosome indicated. An unknown location in the anole genome is indicated as Un

**Fig. 2:**
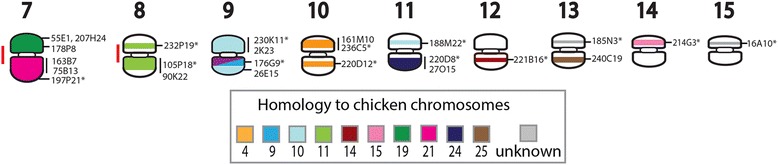
Dragon autosomal microchromosomes. Chicken-dragon comparative map of the nine autosomal microchromosomes. The position of BAC clones is indicated on the *right* of each chromosome, with BACs from the first generation map indicated by an *asterisk*. Chromosomes have been colour-coded to indicate homology to chicken chromosomes. The position of interstitial telomere signals and site of potential fusion is indicated by a *red line* to the *left* of chromosomes 7 and 8. BAC clones assigned to microchromosomes as part of this study were mapped together with anchor BACs that distinguish each of the individual microchromosomes [11]

Our initial approach was to isolate BAC clones containing a gene located at the end of either anole-chicken or chicken-human homologous synteny blocks (HSBs) with the intention that, by mapping a gene from each block, we would be able to extrapolate and assign a virtual location for all genes within the block to the chromosome from which the BAC mapped. A similar approach was successfully used to anchor the tammar wallaby genome to chromosomes, where conserved gene blocks ranged in size from 30 kb to 218 Mb [4, 37, 38]. We chose 40 of the larger 256 anole-chicken HSBs representing 18 chicken chromosomes as well as the six anole macrochromosomes and four anole linkage groups (Additional file 1). BAC clones were mapped to dragon metaphase chromosomes using fluorescent in situ hybridisation (FISH) (for BAC information, see Additional file 1). We also identified human-chicken HSBs in order to cover regions unanchored in the anole genome assembly, particularly the microchromosomes, and mapped 15 of these to dragon chromosomes. BAC clones were end-sequenced to determine their corresponding location in the dragon genome sequence assembly. These data were then added to the existing dragon cytogenetic map [11]. BAC clones mapped and end-sequenced as part of other unpublished studies were also included on this map to bring the total number of 131 BACs mapped to dragon macrochromosomes (Table 1; Fig. 1) and 43 BACs mapped to microchromosomes (Table 2; Fig. 2). Each dragon microchromosome can be distinctly identified by at least one anchor BAC [11]. Using homology of dragon microchromosome anchor BACs with chicken microchromosomes as a guide, 11 of the 13 BACs representing HSBs mapping to microchromosomes were assigned to a specific dragon microchromosome (Fig. 2). Synteny of microchromosomes was conserved between dragon and chicken.

**Table 1:** Estimated portion of genome sequence anchored to dragon macrochromosomes

Chromosome	Number of BACs mapped previously (Young et al, [11])	Number of BACs mapped in current study	Total number of BACs mapped	Sequence anchored (Mb)	Percent of chromosome anchored (%)
1	15	19	34	221.1	68.3
2	14	20	34	117.3	40.5
3	8	12	20	104.9	44.1
4	5	8	13	87.9	41.4
5	7	7	13	63.2	35.5
6	14	3	17	70.1	58.7
Total	63	69	131	664.5	49.0

**Table 2:** Estimated portion of genome sequence anchored to dragon microchromosomes

Chromosome	Number of BACs mapped previously [11, 32]	Number of BACs mapped in current study	Total number of BACs mapped	Sequence anchored (Mb)	Percent of chromosome anchored (%)
7	1	4	5	10.7	20.0
8	2	0	2	8.9	18.5
9	2	2	4	15.0	32.9
10	2	1	3	5.8	13.3
11	2	1	3	16.1	38.7
12	1	0	1	0.7	1.5
13	1	1	2	2.5	1.6
14	1	0	1	7.7	21.8
15	1	0	1	1.4	4.4
Z	10	2	12	8.7	25.4
Unknown	2	7	9	7.3	-
Total	25	18	43	84.8	20.6

Mapping of two BACs for 13 different sequence scaffolds tested the accuracy of the genome assembly, with BACs for 10 of these scaffolds supporting the accuracy of the assembly. However, three scaffolds (scf000004, scf000024, scf000112) highlighted potential assembly errors as BACs corresponding to different regions of these scaffolds mapped to different chromosomes.

**Fig. 3:**
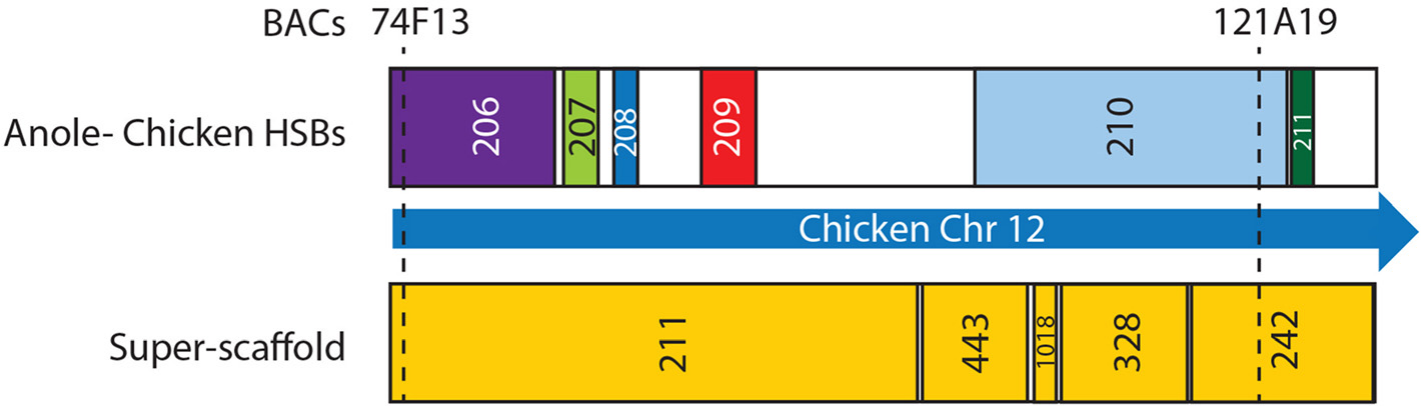
Comparison of sequence assigned by HSBs vs super-scaffold approaches. One super-scaffold (211-443-1018-328-242) with homology to chicken chromosome 12 spans a region covered by six anole-chicken HSBs. Mapping of BACs 74F13 and 121A19 anchor only two of the anole-chicken HSB, leaving large portions of sequence unassigned

We generated 364 super-scaffolds from the dragon genome sequence, joining scaffolds containing contiguous genes when compared to the chicken and anole genomes, assuming that regions displaying conserved synteny between chicken and anole are likely to be conserved as a region in dragon. The super-scaffolds ranged in size from the 302,071 to 23,129,095 bp. The size of the super-scaffolds was determined by adding the size of the individual scaffolds making up a super-scaffold. For example, super-scaffold 15-3-1966 located on chromosome 6 (Table 3) covers a total of 19,301,961 bp because scf000015 is 7,401,090 bp, scf000003 is 11,886,202 bp and scf001966 is 14,669 bp in length. This approach enabled much larger regions of sequence to be assigned to chromosomes than the HSB approach. An example is given in Fig. 3 of a super-scaffold (211-443-1018-328-242) containing orthologues of chicken chromosome 12 and anole chromosome 2 genes. The large anole-chicken HSB_210 (2,266,433 bp) enabled scf000443, scf001018, scf000328 and scf000242 to be linked while the shared synteny between anole and chicken linked scf000211 and scf000443. Mapping of BACs 74F13 and 121A19 would only have assigned two HSBs (HSB_206 and HSB_210) to dragon chromosome 2 whereas the super-scaffolding approach enabled four additional HSBs to be given a chromosomal assignment.

**Table 3:** Supers-scaffolds anchored by two or more BACs

Super-scaffold composition (scaffolds included in super-scaffold)	Size (bp)	Chromosome
**68** - **113**-200-108-923-55-1162-922-**134**	22,504,095	3
**15** - **3**-1966	19,301,961	6
**18**-87-493-387-832-828-874-**67**	19,003,787	4
79-**25**-104-**132**	17,253,217	3
151-459-**57**-737-**101**-624-655-**336**	15,792,258	1
**7** - **525**	10,689,092	11
**211**-443-1018-328-**242**	7,545,615	2

Anchored scaffolds are indicated in bold and underlined

BAC clones unique to several of the largest super-scaffolds that had not previously been localized, either by the HSB approach described above or in other studies, were also isolated and mapped. Eleven super-scaffolds with sequence scaffolds assigned to chromosomes by two or more BACs were used to determine the validity of the super-scaffolding approach (Table 3). Seven super-scaffolds, including the largest one, were supported by mapping data. However, four super-scaffolds had BACs mapping to different chromosomes, indicating rearrangements have occurred since the divergence of anole and dragon from a common ancestor. This means there is either a derived arrangement in dragon, or there are potential assembly errors.

By using the size of the scaffold and, where appropriate, the size of the super-scaffolds, in conjunction with the predicted size of each chromosome [11], we were able to estimate the amount of genome sequence assigned to each chromosome (Tables 1 and 2). Overall, approximately 42 % of the genome sequence was assigned to chromosomes using the super-scaffolding approach.

The super-scaffolding has provided an innovative approach to anchor a large portion of genome sequence to chromosomes for a genome assembly consisting of hundreds of thousands of sequence scaffolds. By doubling the number of BAC clones mapped to chromosomes since the first generation map [11], and integrating BAC end-sequence with the genomic sequence, we physically anchored approximately 42 % of the genome to each of the 16 chromosome pairs. As a comparison, 405 BACs were required to anchor 60 % of the anole genome assembly to chromosomes, yet this resulted in sequence being anchored to only half the microchromosomes [18]. However, our super-scaffolding approach is not without limitations, as we assume that there have been no rearrangements within these super-scaffolds in the dragon lineage. Our results indicate that this assumption is likely to be true for the majority of super-scaffolds but not all. We also have no information on gene order within super-scaffolds, only an assumption that gene content has been conserved. Although the super-scaffolding approach provides an efficient, cost-effective means of assigning sequence to chromosomes, it is important to keep in mind the limitations of this approach when interpreting the data.

### Comparative maps of the dragon autosomes

Comparative maps of each dragon macrochromosome were constructed by comparing the location of mapped scaffolds or super-scaffolds on dragon chromosomes to their corresponding location in the anole and chicken genomes. Each of the dragon macrochromosomes was, for the most part, homologous to the same numbered macrochomosome in the anole lizard, meaning that dragon chromosome 1 was homologous to anole chromosome 1 and so forth. However, intrachromosomal rearrangements were detected on all macrochromosomes except chromosome 2. More importantly, there were 11 interchromosomal rearrangements detected between these two species, distributed across five macrochromosomes (Fig. 1). This is a conservative number as substantial proportions of both genomes remain unanchored.

The lack of intrachromosomal rearrangements detected between dragon and anole chromosome 2 is an interesting observation. The short arm and part of the long arm (just below the centromere) share homology with the chicken Z chromosome (Fig. 1). This region appears to have been conserved largely as an intact region for over 500 million years [39]. Furthermore, the order of up to six chicken Z genes (*ATP5A1, GHR, CHD1, DMRT1, RPS6, ACO1*) is basically conserved between turtle (*Pelodiscus sinensis*), crocodile (*Crocodylus siamensis*), representative squamates (*G. hokouensis, L. reevesii rubritaeniata, E. quadrivirgata, A. carolinensis. V. salvator macromaculatus, P. vitticeps*) [12, 13, 27, 40, 41] and even palaeognathous birds such as the ostrich (*Struthio camelus*) and elegant crested tinamou *(Eudromia elegans)* [42]. This suggests that this gene order may have been present in the common ancestor of birds and reptiles [40]. There may be a feature of this chromosome, beyond its role in sex determination in birds, that has made it less susceptible to rearrangement. However, it is important to keep in mind the limitations of drawing conclusions from the mapping of only several genes between species. It is also important to consider the possibility that the assignment of more sequence to either the dragon or anole chromosome 2 may reveal intrachromosomal rearrangements.

There was limited scope for comparison of dragon and anole microchromosomes as sequence has only been anchored to six of the 12 anole microchromosomes. All six anole microchromosomes share homology with dragon microchromosomes. The ancestral Iguanian karyotype is predicted to have consisted of 12 pairs of microchromosomes as is observed in the anole lizard. The dragon arrangement of ten pairs has been hypothesized to be the result of fusion of microchromosomes giving rise to the reduced number [35]. The presence of interstitial telomere signals on dragon microchromosomes 7 and 8 supports this hypothesis [11]. Our mapping data suggests the fusion of a microchromosome containing chicken chromosome 21 orthologues and presumably a microchromosome with homology to chicken chromosome 19 (the location of orthologues of chicken 19 genes has not been determined in anole). The other fusion is possibly between two microchromosomes (anole chromosomes 7 and 8) with homology to chicken chromosome 11.

Previously, the dragon-chicken comparative map had reported dragon microchromosomes 8 and 11 sharing homology with chicken macrochromosomes [11]. This was based on BLASTN searches of dragon BAC-end sequences against the anole genome. We have shown that this is incorrect and that all but one microchromosome in dragon share homology with chicken microchromosomes. The exception is dragon chromosome 10, which has homology to chicken 4p. Interestingly, chicken 4p is predicted to have been a microchromosome in the amniote ancestor [5].

### Reconstruction of ancestral squamate macrochromosomes

By comparing the dragon-anole-chicken comparative maps to the molecular cytogenetic data available for five other squamates [12–15], we reconstructed the events leading to the squamate, toxicoferan and iguanian ancestral macrochromosome arrangements.

**Fig. 4:**
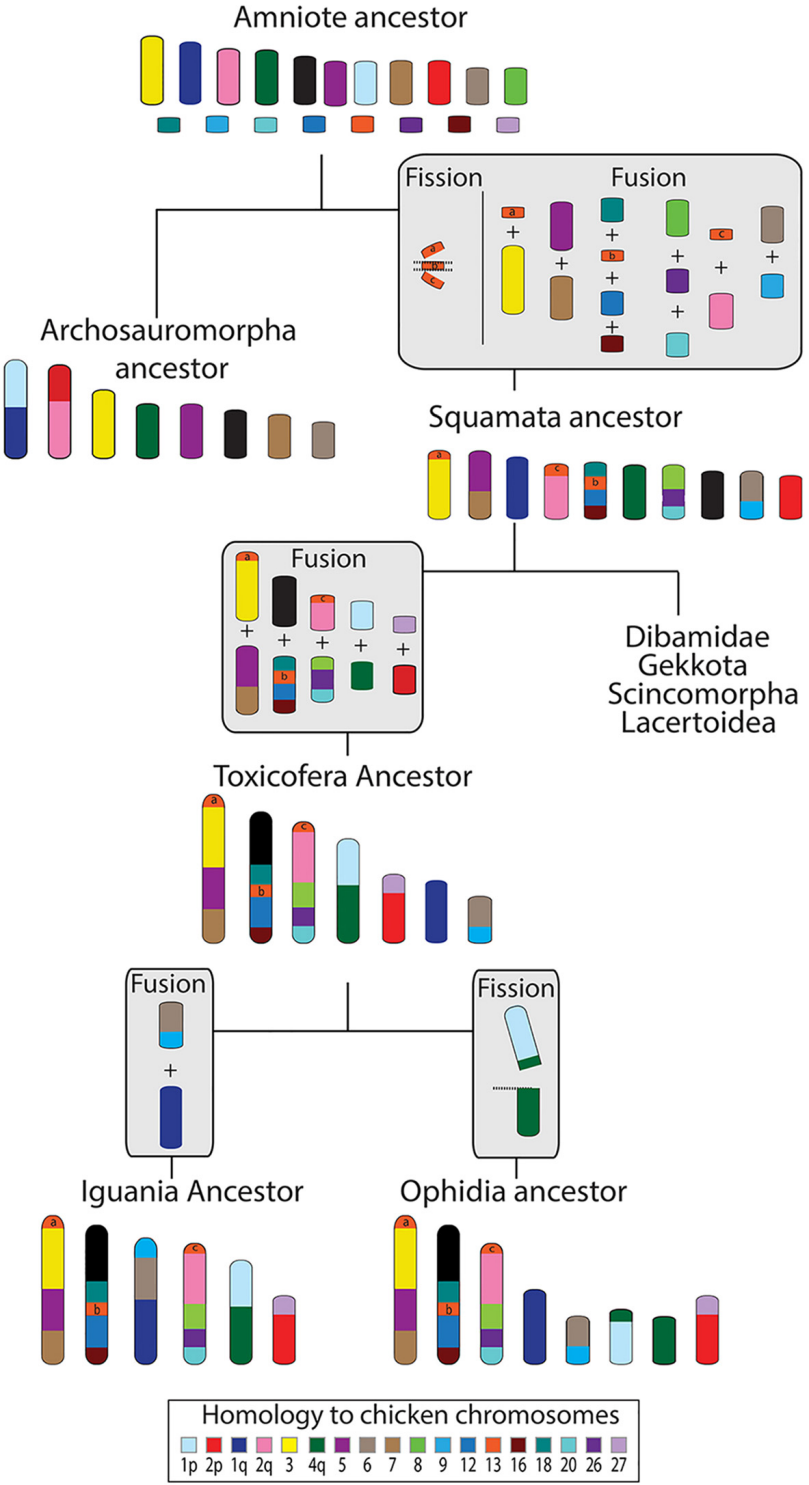
Reconstruction of the Squamate ancestral macrochromosomes. The predicted karyotype for the Amniote and Archosauromorpha (crocodiles, dinosaurs and birds) ancestor is based on Uno et al. [5]. Only microchromosomes relevant to squamate macrochromosome evolution have been included. Boxes in grey indicate the events (fissions or fusions) leading to the predicted karyotype for the Squamata, Toxicofera, Iguania and Ophidia ancestors. The reconstructed chromosomes have been colour-coded for homology to chicken chromosomes

Uno et al. [5] predicted the composition of the ancestral amniote macrochromosomes based on comparisons of extant representatives from different vertebrate lineages. The ancestral amniote karyotype was predicted to consist of 11 pairs of macrochromosomes and at least 14 pairs of microchromosomes [5]. Integration of our new data with these studies suggests that a microchromosome, homologous to chicken 13, fissioned into three fragments that subsequently fused with other chromosomes. Several other rearrangements are common to all squamates (Additional file 2), and suggest the common ancestor had a karyotype consisting of 10 pairs of macrochromosomes as shown in Fig. 4 and an unknown number of microchromosomes. Additional file 2 demonstrates the derivation of the ancestral squamate macrochromosomes. For instance, one part of the fissioned microchromosome sharing homology with chicken chromosome 13 is present on the same chromosome as ancestral amniote chromosome 1 (AnAmn 1) in the Hokou gecko, sand lizard, Japanese four-striped snake, water monitor lizard and anole (genes from this region have not been mapped in dragon or butterfly lizard), suggesting that they fused in the ancestral squamate. Similarly, genes corresponding to AnAmn 6 and AnAmn 8 are located on the same chromosome in all squamate species for which there is cytogenetic mapping data, suggesting the fusion of these two chromosomes in the ancestral squamate.

Comparative cytogenetic mapping from seven squamate species indicates that at least five more fusion events characterise the Toxicofera, to give rise to an ancestor with seven macrochromosomes (Fig. 4). Four of the five rearrangements involved the fusion of two macrochromosomes (see Additional file 2). The fifth fusion was between a microchromosome homologous to chicken chromosome 27 and a macrochromosome homologous to the short arm of chicken chromosome 2. Genes orthologous to chicken chromosomes 2 and 27 are located on the same chromosome in all Toxiferan species studied to date but are on separate chromosomes in the Hokou gecko and sand lizard (Additional file 2). Subsequently, fusion of a macrochromosome sharing homology with chicken 1q and a macrochromosome homologous to chicken chromosomes 6 and 9, gave rise to an iguanian ancestor with six macrochromosomes, similar to those observed in dragon*,* anole and butterfly lizard (Fig. 4; Additional file 2)*.* The highly conserved snake karyotypes, usually consisting of eight pairs of macrochromosomes [29], including the Z, can be derived by fission of the toxicoferan ancestral macrochromosome that shared homology with the short arm of chicken 1 and the long arm of chicken chromosome 4 (Fig. 4; Additional file 2).

Previously, Srikulnath et al. [12], proposed a sequence of events leading to the rearrangements observed between members of the Toxicofera clade: butterfly lizard, water monitor lizard and Japanese four-striped snake. However, it remained unresolved whether the butterfly lizard chromosome 3 was derived from a fusion of two macrochromosomes corresponding to monitor lizard chromosomes 5 and 6 and snake chromosomes 4 and 5 or whether the reverse was true and there had been a fission event to give rise to the two chromosomes in the monitor lizard and snake. We are able to show from comparsions of cytogenetic mapping data across squamates that there was most likely a fusion event in the iguanian ancestor, giving rise to the configuration for chromosome 3 in butterfly lizard, dragon and anole (Additional file 2). This same study could also not distinguish whether a fusion or fission event was responsible for genes homologous to butterfly lizard chromosome 5 and water monitor lizard chromosome 3 being split among two chromosomes in the Japanese four striped snake [12]. The most parsimonious explanation from our analysis, based on the phylogeny of Toxicofera presented in Additional file 2, is a fission event in the ophidian ancestor (Fig. 4; Additional file 2)*.* In the gecko and sand lizard, genes homologous to chicken 1p and 4q are on separate chromosomes but in all toxicoferan species with available data, at least some chicken 4q genes, if not all, are located on the same chromosome as chicken 1p genes. This suggests that there was a fusion of these two chromosomes in the toxicoferan ancestor. It appears there has been a fission in the Japanese four-striped snake to distribute these genes across two chromosomes, leaving the snake chromosome 6 with chicken 1p and a small region homologous to chicken 4q and snake chromosome 7 homologous to the remainder of chicken 4q (Additional file 2).

These first squamate macrochromosome reconstructions give context for finer scale analysis of rearrangements between species. From comparative maps, it is obvious that there are many differences between species, even though gene content of macrochromosomes is largely conserved. Examining the evolutionary history of microchromosomes in these species would be similarly interesting, particularly because it appears that the fusion of microchromosomes, either with macrochromosomes or other microchromosomes, often leads to the differences in karyology observed between species. For example, the fusion of microchromosomes, either to each other and/or to macrochromosomes, accounts for the reduction in microchromosome number in the dragon from that observed in the iguanian ancestor, the greatly reduced number of microchromosomes in the sand lizard [14], and the absence of microchromosomes in the Hokou gecko [15]. Unfortunately, assignment of genes or sequence to specific microchromosomes for most squamates is lacking at this stage. It is crucial for future work to focus on the gene content of microchromosomes if we are to gain a more detailed understanding of squamate karyotype evolution.

### Anchoring sequence to the sex chromosomes

**Fig. 5:**
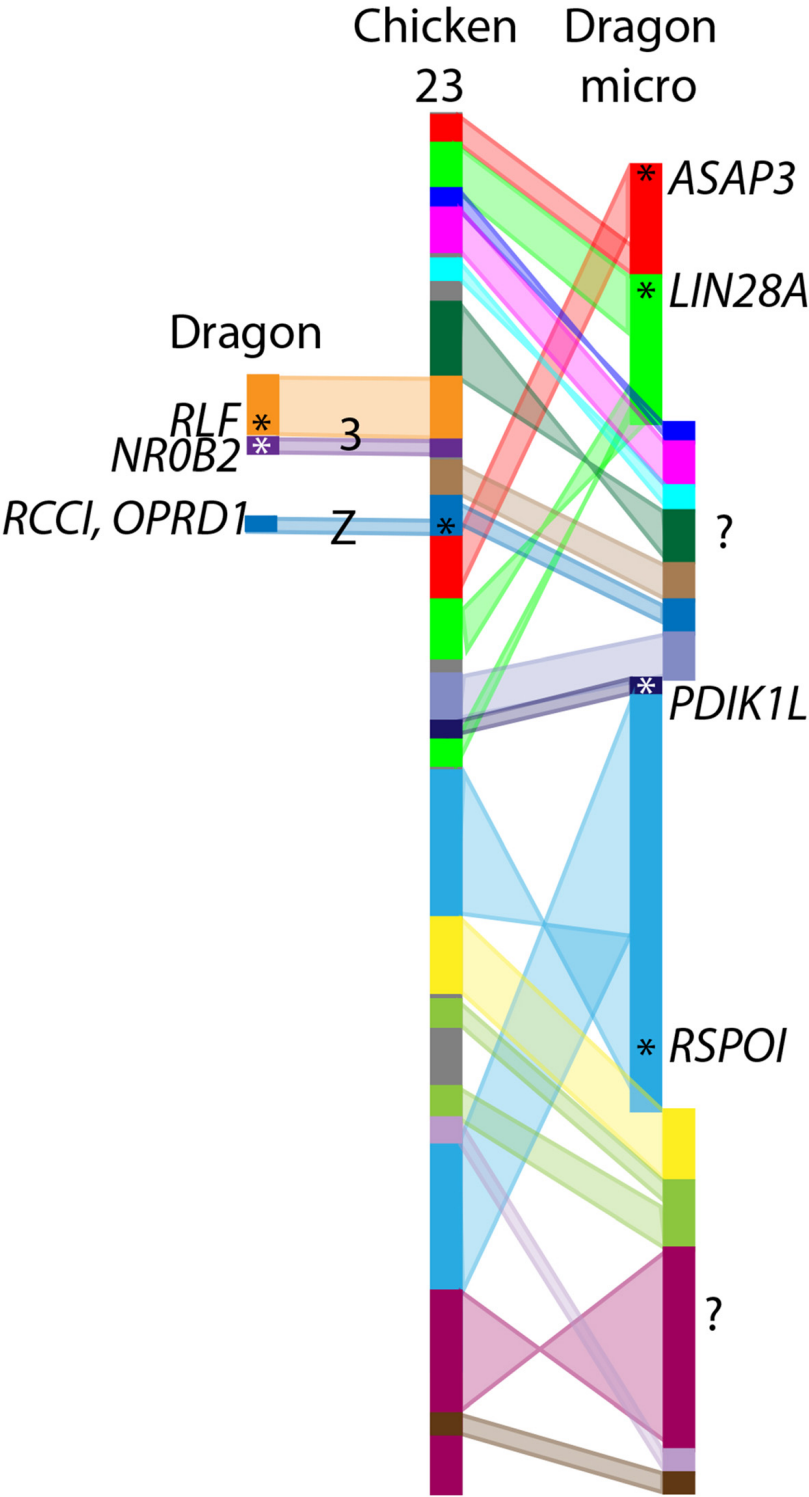
Dragon genome scaffolds with homology to chicken chromosome 23. Each dragon scaffold is indicated in a different colour and the arrangement of these scaffolds on chicken chromosome 23 is shown. One scaffold maps to the dragon Z chromosome, two map to chromosome 3, four map to the same microchromosome and the remainder are unmapped (indicated by ?). *Asterisks* mark the location of genes within a scaffold that have been mapped

Previously, 352 kbp of sequence had been assigned to dragon sex chromosomes, which included no genes with a known role in sex determination or differentiation pathways [32]. Hence, an important reason behind anchoring sequence to dragon chromosomes was to assign more sequence to the sex chromosomes and identify potential sex determining genes. Ezaz et al. [32] assigned several BAC clones to dragon sex chromosomes that contained two genes (*RCC1* and *OPRD1*) whose orthologues map to chicken chromosome 23. However, a third gene whose orthologue maps to chicken chromosome 23, *RSPO1,* is autosomal in the dragon. In the dragon genome assembly, orthologues of genes from chicken chromosome 23 are spread across 16 genome scaffolds. We mapped BACs corresponding to five of these scaffolds, with two mapping to chromosome 3 (scf000752, 0.5 Mb; scf001301, 0.1 Mbp) and three (scf000345, 1.5 Mb; scf000458, 1.1 Mbp; scf001437, 0.6 Mbp) mapping to the same microchromosome as *RSPO1* (scf000275, 1.9 Mbp) (Fig. 5). To date, the only genes orthologous to chicken chromosome 23 confirmed to be on the dragon sex chromosomes are the two originally reported [32], *RCC1* (scf000777, 0.5 Mbp) and *OPRD1* (scf002443, 0.06 Mbp). This means that only a small fragment of the genome orthologous to chicken chromosome 23 is on the dragon sex chromosomes, leaving the rest of the gene content of the sex chromosomes unresolved.

**Fig. 6:**
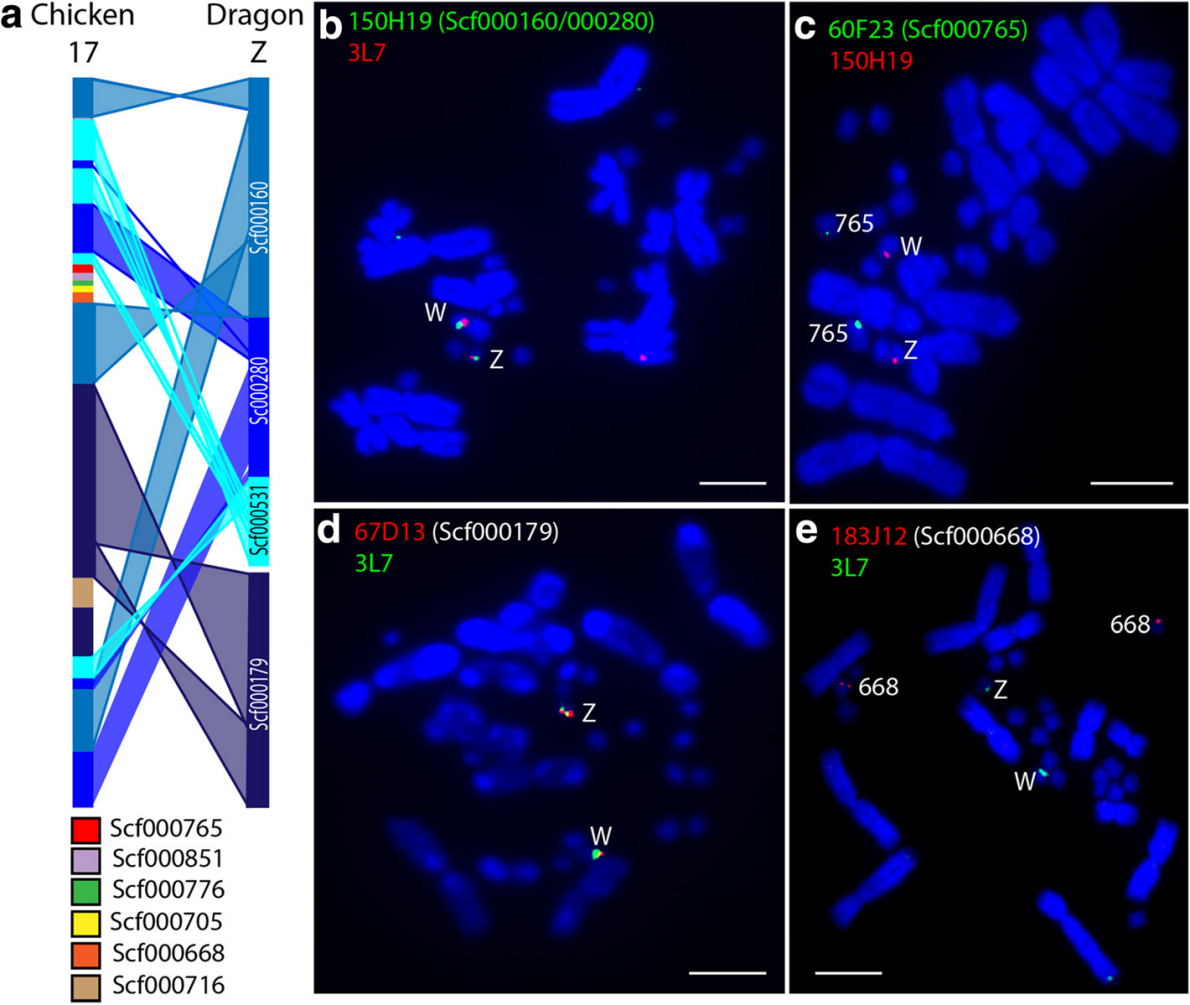
Dragon scaffolds with homology to chicken chromosome 17. **a** Comparison of gene order on chicken chromosome 17 and four scaffolds on dragon Z chromosome (the precise position and orientation of scf000179 is unknown). Mapping of BACs corresponding to Z scaffolds scf000160 and scf000280 (**b**) and scf000179 (**d**) with the BAC 3L7, previously mapped to dragon sex chromosomes [32] onto female metaphase chromosomes. Scf000765 (**c**) and scf000668 (**e**) do not map to the sex chromosomes. Scale bars indicate 10 μm

From the list of scaffolds located on microchromosomes, we identified three scaffolds containing genes involved in the sex differentiation pathway: *WNT4* (scf000491)*, CYP19A1* (scf000121) and *NR5A1* (scf000160). The scaffold containing *CYP19A1* (BAC 26E15) was identified as being located on microchromosome 9 (Fig. 2). The BACs corresponding to the other two scaffolds were used in FISH experiments with a BAC (3L7) previously mapped to dragon sex chromosomes [32]. The *WNT4* scaffold BAC (163B17) mapped to an autosomal microchromosome (Additional file 3) whereas the *NR5A1* scaffold BAC (150H19) was localized to the sex chromosomes (Fig. 6). This scaffold shares homology with chicken chromosome 17. We then proceeded to identify all scaffolds with homology to chicken chromosome 17. BAC end sequence for 150H19 connects Scf000160 (2.89 Mb) and scf000280 (1.87 Mbp). Scf000179 (2.67 Mbp) is assigned to the sex chromosomes by BAC 67D13 (Fig. 6), bringing the total amount of new sequence assigned to the Z chromosome to 7.43 Mbp and consisting of at least 183 genes. Furthermore, exons of the gene *CNTRL* link scf000280 to unmapped scf000531 (0.91 Mbp, 36 genes), which also shares homology with chicken chromosome 17. The 3’ exons of *CNTRL* are located at the end of scf000280 and the 5’ exons are at the start of scf000531, suggesting that these two scaffolds co-locate on the sex chromosomes in dragon*.* This brings the total amount of sequence assigned by these four scaffolds to the Z chromosome to 8.34 Mbp and at least 219 genes. Therefore, our proposed order of scaffolds on the Z chromosome is scf000160 followed by scf000280 and scf000531 but we cannot determine whether scf000179 precedes scf000160 or follows scf000531. In all cases, BACs corresponding to sex chromosome scaffolds mapped to both the Z and W chromosomes in females (Fig. 6).

The sex chromosomes, therefore, consist of a small fragment homologous to chicken chromosome 23 and the rest is homologous to chicken chromosome 17. It would seem that there has been a transposition of this small region of chicken 23 genes to the microchromosome homologous to chicken 17. As *RCCD1* and *OPRD1* are surrounded by zinc finger protein genes, as well as containing a high proportion of repetitive sequences [32], it is possible that this transposition is the result of an illegitimate recombination event.

Although the four sex chromosome scaffolds account for a large proportion of chicken 17, not all scaffolds containing chicken 17 genes map to dragon sex chromosomes, with scf000668 (0.63 Mbp) and scf000765 (0.50 Mbp) mapping to two different pairs of microchromosomes (Fig. 6). There are a further four small scaffolds containing chicken chromosome 17 genes that are yet to be assigned to chromosomes. Three of these scaffolds fall within a region orthologous to chicken chromosome 17 that is flanked by the two autosomal scaffolds, suggesting that this entire segment may be distributed on autosomes in dragon.

A comparison of gene arrangement on the dragon sex chromosome scaffolds and their orthologues in chicken indicates that a substantial number of rearrangements have occurred in this region (Fig. 6). It is impossible at this stage to compare the gene arrangement of these scaffolds with that of anole as this region is spread over more than 25 scaffolds in the anole assembly, the majority of which have not been assigned to chromosomes.

Our assignment of four genome scaffolds to the Z chromosome has provided a list of over 200 genes whose role in sex determination can be assessed. One gene that stands out among this list is *NR5A1* (nuclear receptor subfamily 5, group A, member 1) because it has a known role in the sex determination and differentiation pathways in vertebrates. The expression of this gene has been noted to be highly changeable throughout vertebrate evolution, displaying expression patterns compatible with a role in testis development, ovarian development or a general role in gonadal development [43]. In humans, mutations in the *NR5A1* gene have been discovered in XY sex reversed individuals [44] and *NR5A1* knockout mice have a sex reversed phenotype [45]. Thus, *NR5A1* is a strong candidate for being the sex determining gene in the dragon and warrants further investigation of its role in dragon sex determination.

## Conclusions

Tracing the evolutionary history of reptile genomes requires comparisons of genome organisation to be made, which can only be performed if genomic sequence is anchored to chromosomes. At present, this remains the limiting step in studies of comparative genome organisation. We have devised a strategy to greatly increase the amount of sequence assigned to chromosomes by creating super-scaffolds based on conserved synteny. Anchoring of the dragon genome sequence to chromosomes has provided an important second anchored squamate genome assembly for comparative genomic studies. Our comparative analysis permitted the reconstruction of chromosomal rearrangements, predominantly fusions, at key positions in squamate evolution. Importantly, we have assigned sequence to all dragon chromosomes, including the sex chromosomes and identified *NR5A1* as a candidate sex determining gene in dragon.

## Methods

### Identification of HSBs and primer design

We identified all one-to-one orthologous genes in anole-chicken or human-chicken HSBs as defined in the Ensembl database (v69, [46]). For each exon (at least 100 bp long) of these orthologous genes, we identified the reciprocal best-hit region in the dragon male and female genomes and only included this region for downstream processing if male and female genome sequences were at least 97 % identical. Primers were then designed to amplify reciprocal best-hit regions using the Primer3 software [47]. We assessed primer pairs using re-PCR software [48] to ensure that primer pairs were specific against the male and female genomes.

### Super-scaffolding

Tables of chicken genes in physical order on the chromosomes were matched, as reference, against their homologues in dragon with their respective assembled scaffolds (Additional file 4). A similar table was generated for anole as reference against dragon (Additional file 5). Dragon scaffolds that abutted with respect to gene order with their junction spanned by a contiguous series of consecutive genes in chicken and anole, respectively, were joined to form putative derived 2-scaffolds. Orphaned terminal genes presented a problem, because they linked in both directions when they were sandwiched between the terminal genes of two other scaffolds. In instances where the opposite terminal to the orphan (on the same scaffold) also supported joining, the chaining of scaffolds forked. This was overcome by skipping orphaned terminal genes when joining scaffolds. Scaffolds with single genes were retained (refer to Additional file 6). The 2-scaffolds were filtered for those that were supported by consensus between chicken and anole (refer to Additional file 7). Finally, the 2-scaffolds generated by consensus were assembled into putative super-scaffolds (refer to Additional file 8).

Primers to isolate BACs for unmapped super-scaffolds were designed by firstly masking repeat sequences using Repbase [49]. Primer3 software [47] was then used to design primers to unmasked regions and the specificity of these primers was confirmed by performing a BLASTN search against the *P.vitticeps* male genome [34]. All primers used for library screening are listed in Additional file 1.

### BAC library screening

Primers were used to screen the *P. vitticeps* PCR Bacterial Artificial Chromosome (BAC) library (6.2x, Amplicon Express, Pullman, WA, USA) following the manufacturer’s instructions. Briefly, for each primer pair, there were two rounds of PCR BAC library screening. The first round included an initial screen of superpools, to identify subsequent matrixpools for screening. The second round included screening of matrixpools to identify respective BAC clones containing the candidate genes. Primers (0.32 μM) were added to 1x MyTaq™ HS Red Mix (Bioline Australia Pty Ltd, Alexandria, NSW, Australia) and corresponding BAC library DNA (2 μl; superpool/matrixpool), and cycled with the following conditions; 95 °C, 5 mins; (95 °C, 30 s; primer annealing temperature, 30 s; 72 °C, 90 s) x 35; 72 °C, 10 mins. PCR products were analysed on a 1 % agarose (Amresco LLC, Solon, OH, USA) gel.

All BACs were end sequenced with vector primers T7 (5’-TAATACGACTCACTATAGGG-3’) and/or pCC1/pEpiFOS-5 reverse primer (5’-CTCGTATGTTGTGTGGAATTGTGAGC-3’) by Macrogen Inc. (Seoul, South Korea) and sequences were submitted to NCBI GSS (dbGSS KS332314 - KS332403). BLASTN searches were performed with the BAC end sequences in order to determine the location of each BAC in the dragon genome assembly and to confirm the target content of the isolated BACs.

### Fluorescent in situ hybridization

BAC DNA was extracted using the Promega WIZARD plus SV miniprep DNA purification system (Promega, Alexandria, NSW, Australia). Nick translation was used to label approximately 1 μg of BAC DNA with SpectrumOrange or SpectrumGreen dUTP (Abbott Molecular Inc., Des Plaines, IL, USA). Labeled probes were precipitated overnight at –20 °C with 1 μg sheared *P. vitticeps* genomic DNA and 1 μl glycogen. The probes were prepared for hybridization as described by Alsop et al. [50]. Female *P. vitticeps* metaphase chromosomes were prepared from fibroblast cell lines previously established in the lab from samples collected under the approval of the Animal Ethics Committee of the University of Canberra (CEAE 04/4). Metaphase spreads were denatured by placing the slides on a 65 °C heat block. Slides were washed in 0.4x SSC, 0.3 % (v/v) Igepal CA-630 (Sigma-Alrdich Pty Ltd, Castle Hill, NSW, Australia) followed by 2x SSC, 0.1 % (v/v) Igepal CA-630 for 2 min at 60 °C and 1 min at room temperature, respectively and mounted with DAPI in vectashield (1:3; Vector Laboratories Inc., Burlingame, CA, USA). Slides were examined using a Zeiss Axio Scope A1 epifluorescence microscope fitted with a high-resolution microscopy camera AxioCam MRm Rev. 3 (Carl Zeiss Ltd, Cambridge, UK). At least 10 images were captured for each BAC and Fractional Length from the p terminus (Flpter) values [51] were obtained for BACs mapping to macrochromosomes using MetaSystems ISIS software (MetaSystems, Newton, MA, USA).

## Abbreviations

BAC, bacterial artificial chromosome; BLASTN, basic local alignment search tool nucleotide; FISH, fluorescent in situ hybridisation; Flpter, fractional length from the p terminus; HSB, homolgous synteny block

## Additional files

Additional file 1: HSB, primer and BAC clone information. (XLSX 134 kb) Additional file 2: Comparison of squamate macrochromosomes for ancestral Squamata, Toxicofera and Iguania reconstructions. (PDF 1578 kb) Additional file 3: Localisation of *WNT4* and *CYP19A1* scaffolds to autosomal microchromosomes. (PDF 387 kb) Additional file 4: Gene synteny between chicken and dragon (XLSX 2298 kb) Additional file 5: Gene synteny between anole and dragon (XLSX 2485 kb) Additional file 6: Script A1 to scan the synteny files to interim super-scaffolds for *P. vitticeps. (CGI 22 kb)*
Additional file 7: Script A2 to take output from synteny_pairwise.cgi and simultaneously combine species information. (CGI 9 kb) Additional file 8: Script A3 to chain pairwise super-scaffolds into larger chains. (CGI 8 kb)
