# Supplementary figures and images for: Anchoring genome sequence to chromosomes of the central bearded dragon (Pogona vitticeps) enables reconstruction of ancestral squamate macrochromosomes and identifies sequence content of the Z chromosome
Source: BMC Genomics. 2016 Jun 10;17:447. doi: 10.1186/s12864-016-2774-3 (PMC4902969; doi:10.1186/s12864-016-2774-3)

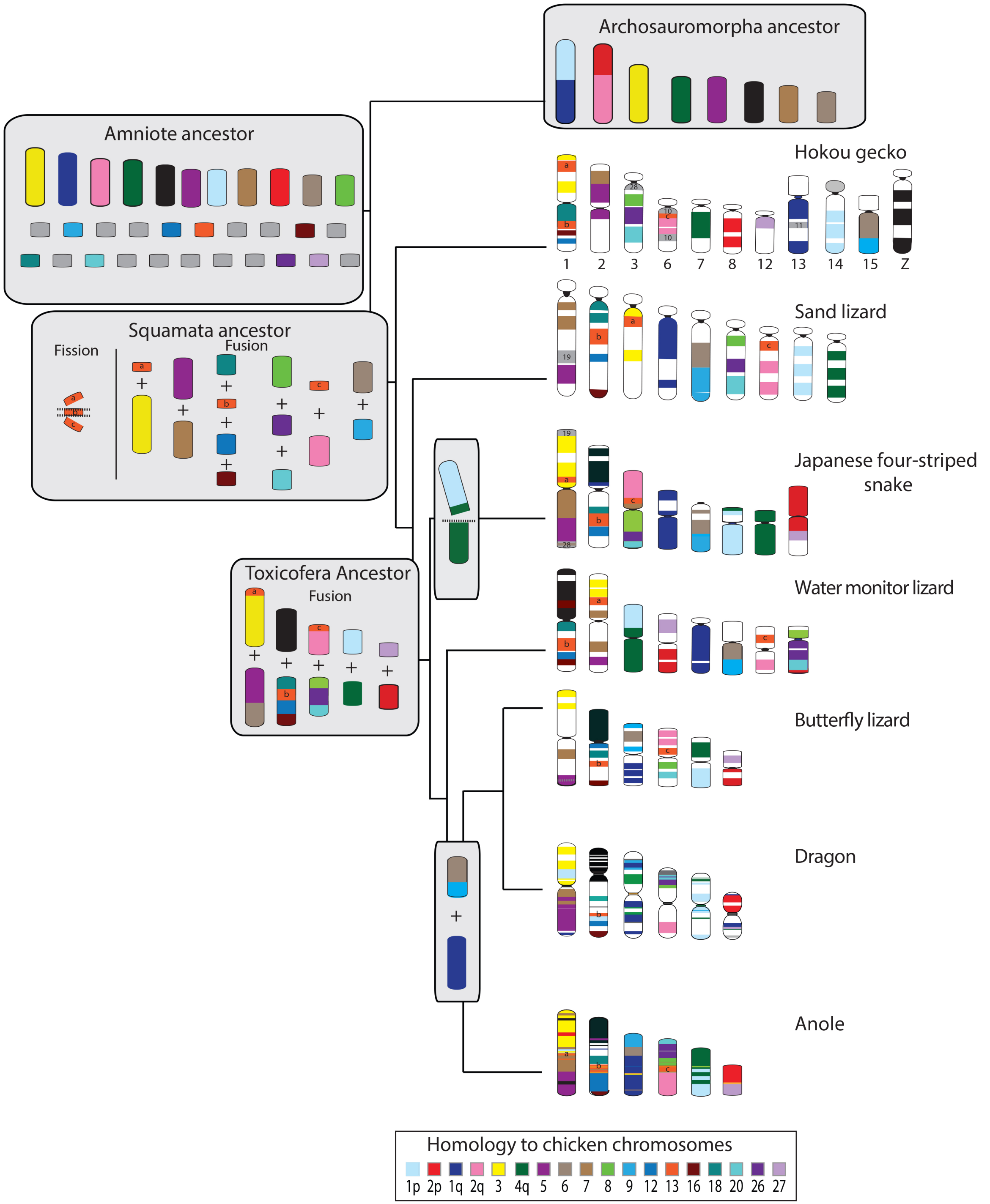

Supplement: Additional file 2: — Comparison of squamate macrochromosomes for ancestral Squamata, Toxicofera and Iguania reconstructions. (PDF 1578 kb) [file 12864_2016_2774_MOESM2_ESM.pdf]

163B17 (*WNT4*)  
3L7 (sex chromosomes)

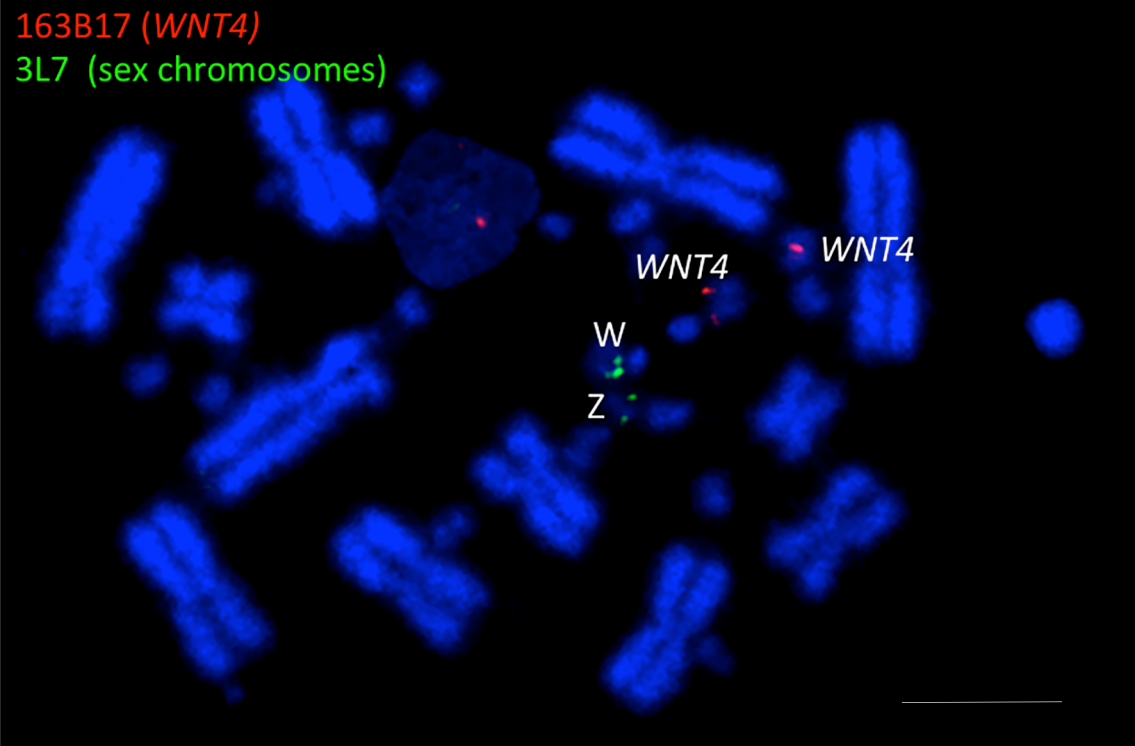

Supplement: Additional file 3: — Localisation of WNT4 and CYP19A1 scaffolds to autosomal microchromosomes. (PDF 387 kb) [file 12864_2016_2774_MOESM3_ESM.pdf]
